# Supplementary material for: Association between oral microbiome and seven types of cancers in East Asian population: a two-sample Mendelian randomization analysis
Source: Front Mol Biosci. 2023 Nov 21;10:1327893. doi: 10.3389/fmolb.2023.1327893 (PMC10702768; doi:10.3389/fmolb.2023.1327893)
Supplement: Supplementary file 1 [file DataSheet1.ZIP › Supplementary Materials/MR plots for tongue/tongue═╝/Ovarian cancer/pheno.2280_to_ovarian cancer_forest.pdf]

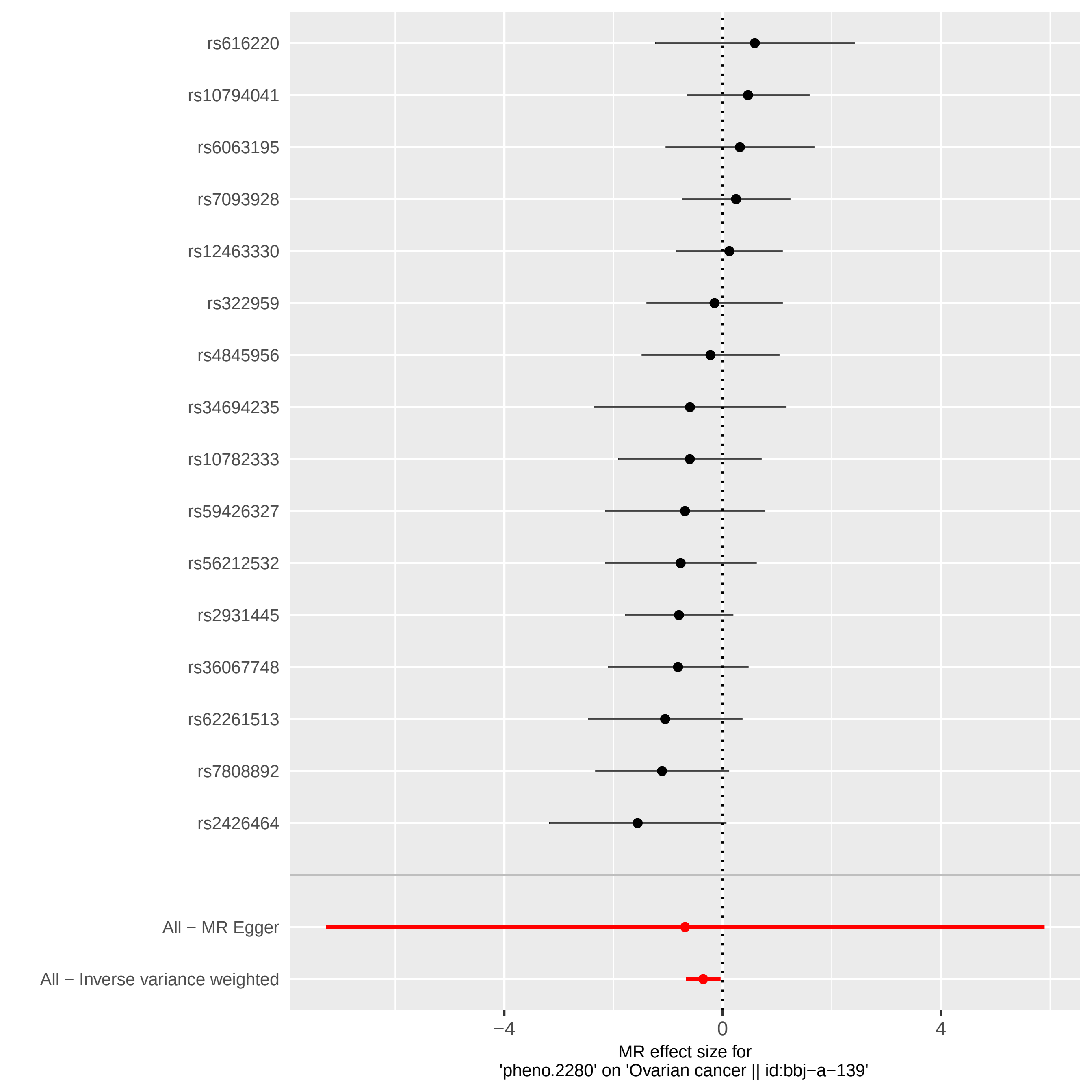

rs616220
rs10794041
rs6063195
rs7093928
rs12463330
rs322959
rs4845956
rs34694235
rs10782333
rs59426327
rs56212532
rs2931445
rs36067748
rs62261513
rs7808892
rs2426464
All − MR Egger
All − Inverse variance weighted
−4
0
4
MR effect size for
'pheno.2280' on 'Ovarian cancer || id:bbj−a−139'
